# Diaqua­dibromidobis[3-dimethyl­amino-1-(4-pyridyl-κ*N*)prop-2-en-1-one]cadmium(II)

**DOI:** 10.1107/S1600536809007028

**Published:** 2009-03-06

**Authors:** Hua-Ze Dong, Zhao-Lian Chu, Nai-Liang Hu

**Affiliations:** a Department of Chemistry and Chemical Engineering, Hefei Teachers College, Hefei 230061, People’s Republic of China; b Institute of Molecular Engineering & Applied Chemistry, School of Chemistry and Chemical Engineering, Anhui University of Technology, Maanshan 243002, People’s Republic of China; c School of Chemistry and Chemical Engineering, Anhui University, Hefei 230039, People’s Republic of China

## Abstract

In the title compound, [CdBr_2_(C_10_H_12_N_2_O)_2_(H_2_O)_2_], the Cd^II^ ion is located on an inversion center and is six-coordinated by two N atoms [Cd—N = 2.377 (3) Å] from two different 3-dimethyl­amino-1-(4-pyrid­yl)prop-2-en-1-one ligands, two O atoms [Cd—O = 2.355 (2) Å] from two coordinated water mol­ecules and two bromide anions [Cd—Br = 2.6855 (5) Å]. Inter­molecular O—H⋯O hydrogen bonds link the mol­ecules into layers parallel to the *bc* plane.

## Related literature

For general backgroud, see: Bi *et al.* (2008 ▶); Dong *et al.* (2008 ▶). For related structures, see: Hu *et al.* (2003 ▶); Ito *et al.* (1984 ▶). For details of the synthesis, see Sun *et al.* (2008 ▶).
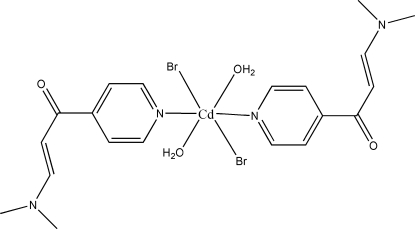


## Experimental

### 

#### Crystal data

[CdBr_2_(C_10_H_12_N_2_O)_2_(H_2_O)_2_]
*M* *_r_* = 660.68
Monoclinic, 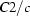

*a* = 21.362 (3) Å
*b* = 8.4360 (9) Å
*c* = 14.6371 (16) Å
β = 114.456 (3)°
*V* = 2401.1 (5) Å^3^
*Z* = 4
Mo *K*α radiation
μ = 4.27 mm^−1^
*T* = 273 K
0.2 × 0.2 × 0.2 mm

#### Data collection

Bruker SMART CCD area-detector diffractometer
Absorption correction: multi-scan (*SADABS*; Bruker, 2000 ▶) *T*_min_ = 0.407, *T*_max_ = 0.424
6227 measured reflections
2356 independent reflections
2085 reflections with *I* > 2σ(*I*)
*R*_int_ = 0.073

#### Refinement

*R*[*F* ^2^ > 2σ(*F* ^2^)] = 0.035
*wR*(*F* ^2^) = 0.090
*S* = 1.02
2356 reflections
144 parameters
H-atom parameters constrained
Δρ_max_ = 0.62 e Å^−3^
Δρ_min_ = −0.93 e Å^−3^

### 

Data collection: *SMART* (Bruker, 2000 ▶); cell refinement: *SAINT* (Bruker, 2000 ▶); data reduction: *SAINT*; program(s) used to solve structure: *SHELXS97* (Sheldrick, 2008 ▶); program(s) used to refine structure: *SHELXL97* (Sheldrick, 2008 ▶); molecular graphics: *SHELXTL* (Sheldrick, 2008 ▶); software used to prepare material for publication: *SHELXTL*.

## Supplementary Material

Crystal structure: contains datablocks I, global. DOI: 10.1107/S1600536809007028/cv2525sup1.cif

Structure factors: contains datablocks I. DOI: 10.1107/S1600536809007028/cv2525Isup2.hkl

Additional supplementary materials:  crystallographic information; 3D view; checkCIF report

## Figures and Tables

**Table 1:** Hydrogen-bond geometry (Å, °)

*D*—H⋯*A*	*D*—H	H⋯*A*	*D*⋯*A*	*D*—H⋯*A*
O2—H2*A*⋯O1^i^	0.85	2.02	2.770 (3)	147
O2—H2*B*⋯O1^ii^	0.85	2.31	2.751 (4)	113

Symmetry codes: (i) 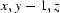; (ii) 
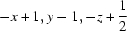
.

**Fig. 1.**
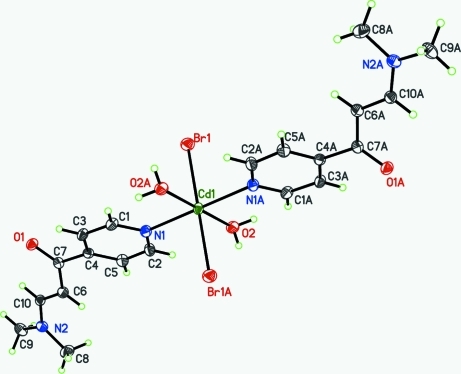
Molecular structure of the title compound, showing 30% probability displacement ellipsoids and the atom-numbering [symmetry code: (A) 1 - x,1 - y,1 - z].

## supplementary crystallographic information

### Comment

In recent years, researchers showed considerable interest in the physical and chemical properties of mono- and polynuclear complexes of transition metals having the d^10^ electronic configuration (Bi *et al.*, 2008; Dong *et al.*, 2008). Ligands with pyridyl group have been used to generate various metal-organic architectures with cadmium salts (Hu *et al.*, 2003; Ito *et al.*, 1984). Here we report a new monomeric cadmium(II) complex, *viz. *the title compound, [Cd(C_10_H_12_N_2_O)_2_Br_2_(H_2_O)_2_].

The asymmetric unit of the title compound contains a half of centrosymmetric molecule, and the Cd^II^ ion lies on an inversion center. Each Cd^II^ ion exhibits an octahedral environment with two nitrogen atoms from the pyridyl groups of two ligands, two oxygen atoms from two coordinated water molecules, and two bromine anions (Fig. 1). Intermolecular O—H···O hydrogen bonds  (Table 1) link the molecules into layers parallel to *bc *plane.

### Experimental

All solvents and chemicals were of analytical grade and were used without further purification. Ligand was prepared by similar procedure reported in the literature (Sun *et al.*, 2008). For the synthesis of title compoud, a solution of ligand (0.1 mmol), CdBr_2 _(0.1 mmol) in 30 ml me thanol was refluxed for 2 h, and then cooled to room temperature and filtered. Single crystals suitable for X-ray analysis were grown from the methanol solution by slow evaporation at room temperature in air. Anal. Calcd. for C_20_H_28_CdN_4_O_4_Br_2_: C, 36.36; H, 4.27; N, 8.48. Found: C, 36.38; H, 4.38; N, 8.32. Main FT—IR (KBr, cm^-1^): 3078(*w*), 1627(*s*), 1603(*m*), 1558(*w*),1498(*s*), 1437(*m*), 1384(*m*), 1329(*w*),1233(*m*),781(*w*).

### Refinement

All hydrogen atoms were geometrically positioned (C—H 0.93–0.97 Å, O–H 0.85 Å) and refined as riding, with *U*_iso_(H)=1.2–1.5 *U*_eq_ of the parent atom.

### Crystal data

**Table:**

[CdBr_2_(C_10_H_12_N_2_O)_2_(H_2_O)_2_]	*F*(000) = 1304
*M**_r_* = 660.68	*D*_x_ = 1.828 Mg m^−^^3^
Monoclinic, *C*2/*c*	Mo *K*α radiation, λ = 0.71073 Å
Hall symbol: -C 2yc	Cell parameters from 3328 reflections
*a* = 21.362 (3) Å	θ = 2.6–27.8°
*b* = 8.4360 (9) Å	µ = 4.27 mm^−^^1^
*c* = 14.6371 (16) Å	*T* = 273 K
β = 114.456 (3)°	Block, colourless
*V* = 2401.1 (5) Å^3^	0.2 × 0.2 × 0.2 mm
*Z* = 4	

### Data collection

**Table:**

Bruker SMART CCD area-detector diffractometer	2356 independent reflections
Radiation source: fine-focus sealed tube	2085 reflections with *I* > 2σ(*I*)
graphite	*R*_int_ = 0.073
φ and ω scans	θ_max_ = 26.0°, θ_min_ = 2.1°
Absorption correction: multi-scan (*SADABS*; Bruker, 2000)	*h* = −26→25
*T*_min_ = 0.407, *T*_max_ = 0.424	*k* = −8→10
6227 measured reflections	*l* = −18→17

### Refinement

**Table:**

Refinement on *F*^2^	Primary atom site location: structure-invariant direct methods
Least-squares matrix: full	Secondary atom site location: difference Fourier map
*R*[*F*^2^ > 2σ(*F*^2^)] = 0.035	Hydrogen site location: inferred from neighbouring sites
*wR*(*F*^2^) = 0.090	H-atom parameters constrained
*S* = 1.01	*w* = 1/[σ^2^(*F*_o_^2^) + (0.0479*P*)^2^] where *P* = (*F*_o_^2^ + 2*F*_c_^2^)/3
2356 reflections	(Δ/σ)_max_ < 0.001
144 parameters	Δρ_max_ = 0.62 e Å^−^^3^
0 restraints	Δρ_min_ = −0.93 e Å^−^^3^

### Special details

**Table:**

Experimental. The structure was solved by direct methods (Bruker, 2000) and successive difference Fourier syntheses.
Geometry. All e.s.d.'s (except the e.s.d. in the dihedral angle between two l.s. planes) are estimated using the full covariance matrix. The cell e.s.d.'s are taken into account individually in the estimation of e.s.d.'s in distances, angles and torsion angles; correlations between e.s.d.'s in cell parameters are only used when they are defined by crystal symmetry. An approximate (isotropic) treatment of cell e.s.d.'s is used for estimating e.s.d.'s involving l.s. planes.
Refinement. Refinement of *F*^2^ against ALL reflections. The weighted *R*-factor *wR* and goodness of fit *S* are based on *F*^2^, conventional *R*-factors *R* are based on *F*, with *F* set to zero for negative *F*^2^. The threshold expression of *F*^2^ > σ(*F*^2^) is used only for calculating *R*-factors(gt) *etc*. and is not relevant to the choice of reflections for refinement. *R*-factors based on *F*^2^ are statistically about twice as large as those based on *F*, and *R*- factors based on ALL data will be even larger.

### Fractional atomic coordinates and isotropic or equivalent isotropic displacement parameters (Å^2^)

**Table:**

	*x*	*y*	*z*	*U*_iso_*/*U*_eq_	
Cd1	0.5000	0.5000	0.5000	0.02740 (13)	
Br1	0.626658 (19)	0.61470 (5)	0.54529 (3)	0.04254 (15)	
C1	0.44435 (18)	0.8564 (4)	0.4098 (2)	0.0332 (8)	
H1	0.4561	0.8760	0.4775	0.040*	
C2	0.43309 (18)	0.6830 (4)	0.2848 (2)	0.0350 (8)	
H2	0.4368	0.5807	0.2639	0.042*	
C3	0.42099 (19)	0.9802 (4)	0.3443 (2)	0.0317 (8)	
H3	0.4171	1.0810	0.3673	0.038*	
C4	0.40298 (16)	0.9534 (4)	0.2423 (2)	0.0270 (7)	
C5	0.40919 (18)	0.7997 (4)	0.2139 (2)	0.0341 (8)	
H5	0.3971	0.7758	0.1467	0.041*	
C6	0.33391 (18)	1.0483 (5)	0.0698 (2)	0.0337 (8)	
H6	0.3149	0.9472	0.0558	0.040*	
C7	0.38011 (16)	1.0865 (4)	0.1687 (2)	0.0276 (7)	
C8	0.2340 (2)	0.9862 (5)	−0.1300 (3)	0.0524 (11)	
H8A	0.2646	0.9016	−0.1281	0.079*	
H8B	0.1990	0.9969	−0.1970	0.079*	
H8C	0.2131	0.9631	−0.0848	0.079*	
C9	0.2647 (2)	1.2464 (5)	−0.1787 (3)	0.0423 (9)	
H9A	0.2856	1.3452	−0.1492	0.063*	
H9B	0.2167	1.2629	−0.2196	0.063*	
H9C	0.2866	1.2058	−0.2195	0.063*	
C10	0.31637 (17)	1.1583 (4)	−0.0065 (2)	0.0299 (7)	
H10	0.3371	1.2575	0.0093	0.036*	
N1	0.45139 (14)	0.7084 (3)	0.38242 (19)	0.0311 (6)	
N2	0.27261 (15)	1.1335 (4)	−0.1000 (2)	0.0338 (7)	
O1	0.40227 (12)	1.2232 (3)	0.19871 (16)	0.0343 (5)	
O2	0.50607 (12)	0.3380 (3)	0.37287 (16)	0.0368 (6)	
H2A	0.4661	0.3344	0.3253	0.044*	
H2B	0.5323	0.3885	0.3525	0.044*	

### Atomic displacement parameters (Å^2^)

**Table:**

	*U*^11^	*U*^22^	*U*^33^	*U*^12^	*U*^13^	*U*^23^
Cd1	0.0341 (2)	0.0237 (2)	0.02374 (19)	0.00164 (13)	0.01137 (15)	0.00238 (13)
Br1	0.0377 (2)	0.0426 (3)	0.0455 (2)	−0.00547 (16)	0.01541 (19)	0.00457 (17)
C1	0.043 (2)	0.031 (2)	0.0228 (15)	−0.0064 (15)	0.0108 (14)	0.0005 (14)
C2	0.043 (2)	0.0280 (19)	0.0297 (16)	0.0049 (15)	0.0112 (15)	0.0012 (15)
C3	0.043 (2)	0.0242 (19)	0.0264 (16)	−0.0044 (14)	0.0129 (15)	−0.0006 (13)
C4	0.0252 (17)	0.0282 (17)	0.0270 (15)	−0.0016 (13)	0.0102 (13)	0.0032 (14)
C5	0.042 (2)	0.036 (2)	0.0241 (15)	0.0019 (15)	0.0131 (15)	−0.0017 (15)
C6	0.037 (2)	0.0299 (19)	0.0284 (16)	−0.0003 (15)	0.0076 (15)	0.0022 (15)
C7	0.0299 (18)	0.029 (2)	0.0260 (16)	0.0017 (14)	0.0132 (14)	0.0028 (14)
C8	0.058 (3)	0.049 (3)	0.037 (2)	−0.0098 (19)	0.006 (2)	−0.0102 (18)
C9	0.044 (2)	0.051 (2)	0.0299 (17)	0.0098 (18)	0.0135 (16)	0.0129 (17)
C10	0.0322 (18)	0.0291 (18)	0.0275 (15)	0.0002 (14)	0.0115 (14)	−0.0019 (14)
N1	0.0342 (16)	0.0294 (17)	0.0295 (14)	−0.0007 (12)	0.0129 (12)	0.0049 (12)
N2	0.0338 (16)	0.0398 (18)	0.0251 (13)	0.0008 (12)	0.0094 (12)	0.0021 (13)
O1	0.0407 (14)	0.0288 (14)	0.0293 (11)	−0.0061 (10)	0.0105 (11)	0.0017 (10)
O2	0.0361 (13)	0.0434 (15)	0.0304 (12)	−0.0041 (11)	0.0132 (10)	−0.0094 (11)

### Geometric parameters (Å, °)

**Table:**

Cd1—O2^i^	2.355 (2)	C6—C10	1.379 (5)
Cd1—O2	2.355 (2)	C6—C7	1.411 (4)
Cd1—N1^i^	2.377 (3)	C6—H6	0.9300
Cd1—N1	2.377 (3)	C7—O1	1.255 (4)
Cd1—Br1^i^	2.6855 (5)	C8—N2	1.455 (5)
Cd1—Br1	2.6855 (5)	C8—H8A	0.9600
C1—N1	1.339 (4)	C8—H8B	0.9600
C1—C3	1.365 (5)	C8—H8C	0.9600
C1—H1	0.9300	C9—N2	1.449 (4)
C2—N1	1.333 (4)	C9—H9A	0.9600
C2—C5	1.366 (5)	C9—H9B	0.9600
C2—H2	0.9300	C9—H9C	0.9600
C3—C4	1.398 (4)	C10—N2	1.316 (4)
C3—H3	0.9300	C10—H10	0.9300
C4—C5	1.385 (5)	O2—H2A	0.8500
C4—C7	1.491 (4)	O2—H2B	0.8501
C5—H5	0.9300		
			
O2^i^—Cd1—O2	180.0	C10—C6—C7	121.2 (3)
O2^i^—Cd1—N1^i^	90.43 (9)	C10—C6—H6	119.4
O2—Cd1—N1^i^	89.57 (9)	C7—C6—H6	119.4
O2^i^—Cd1—N1	89.57 (9)	O1—C7—C6	124.8 (3)
O2—Cd1—N1	90.43 (9)	O1—C7—C4	118.4 (3)
N1^i^—Cd1—N1	180.00 (11)	C6—C7—C4	116.8 (3)
O2^i^—Cd1—Br1^i^	91.41 (6)	N2—C8—H8A	109.5
O2—Cd1—Br1^i^	88.59 (6)	N2—C8—H8B	109.5
N1^i^—Cd1—Br1^i^	90.33 (7)	H8A—C8—H8B	109.5
N1—Cd1—Br1^i^	89.67 (7)	N2—C8—H8C	109.5
O2^i^—Cd1—Br1	88.59 (6)	H8A—C8—H8C	109.5
O2—Cd1—Br1	91.41 (6)	H8B—C8—H8C	109.5
N1^i^—Cd1—Br1	89.67 (7)	N2—C9—H9A	109.5
N1—Cd1—Br1	90.33 (7)	N2—C9—H9B	109.5
Br1^i^—Cd1—Br1	180.000 (15)	H9A—C9—H9B	109.5
N1—C1—C3	123.8 (3)	N2—C9—H9C	109.5
N1—C1—H1	118.1	H9A—C9—H9C	109.5
C3—C1—H1	118.1	H9B—C9—H9C	109.5
N1—C2—C5	123.3 (3)	N2—C10—C6	125.0 (3)
N1—C2—H2	118.3	N2—C10—H10	117.5
C5—C2—H2	118.3	C6—C10—H10	117.5
C1—C3—C4	119.1 (3)	C2—N1—C1	116.8 (3)
C1—C3—H3	120.5	C2—N1—Cd1	120.2 (2)
C4—C3—H3	120.5	C1—N1—Cd1	122.8 (2)
C5—C4—C3	117.0 (3)	C10—N2—C9	121.4 (3)
C5—C4—C7	122.1 (3)	C10—N2—C8	121.3 (3)
C3—C4—C7	120.9 (3)	C9—N2—C8	117.2 (3)
C2—C5—C4	119.9 (3)	Cd1—O2—H2A	107.7
C2—C5—H5	120.0	Cd1—O2—H2B	104.3
C4—C5—H5	120.0	H2A—O2—H2B	108.3

Symmetry codes: (i) −*x*+1, −*y*+1, −*z*+1.

### Hydrogen-bond geometry (Å, °)

**Table:**

*D*—H···*A*	*D*—H	H···*A*	*D*···*A*	*D*—H···*A*
O2—H2A···O1^ii^	0.85	2.02	2.770 (3)	147
O2—H2B···O1^iii^	0.85	2.31	2.751 (4)	113

Symmetry codes: (ii) *x*, *y*−1, *z*; (iii) −*x*+1, *y*−1, −*z*+1/2.
